# Supplementary material for: Rescue of Low-Yield DNA Samples for Next-Generation Sequencing Using Vacuum Centrifugal Concentration in a Clinical Workflow
Source: Reports (MDPI). 2023 May 23;6(2):23. doi: 10.3390/reports6020023 (PMC12225422; doi:10.3390/reports6020023)
Supplement: Supplementary file 1 [file reports-06-00023-s001.zip › reports-2352393-supplementary.pdf]

7. Supplementary

Supplementary Table 1 (S1): Sample concentration, sample volume, and vacuum centrifugation time for Batch 1.

Concentration (ng/µL)	Volume (µL)	Time (minutes)
0.746 (Batch)	55.0	0
0.996	47.0	5
0.980	47.0	5
1.056	44.5	5
1.040	46.0	5
1.088	40.5	10
1.174	39.0	10
1.162	39.5	10
1.118	40.5	10
1.296	32.5	15
1.272	34.5	15
1.302	32.5	15
1.284	34.0	15
1.544	27.0	20
1.550	26.5	20
1.500	27.0	20
1.488	28.0	20
1.542	24.5	25
1.558	24.5	25
1.550	24.5	25
1.538	26.0	25
1.734	17.0	30
1.716	18.0	30
1.780	16.5	30
1.758	17.0	30

Supplementary Table 2 (S2): Sample concentration, sample volume, and vacuum centrifugation time for Batch 2.

Concentration (ng/µL)	Volume (µL)	Time (minutes)
0.170 (Batch)	55.0	0
0.271	42.0	10
0.286	40.0	10
0.239	40.5	10

0.254	41.5	10
0.326	30.0	20
0.356	29.5	20
0.342	28.0	20
0.348	30.5	20
0.518	22.5	30
0.498	19.5	30
0.510	19.0	30
0.620	21.0	30
1.122	11.5	40
1.056	10.0	40
1.000	10.5	40
0.884	11.0	40

Supplementary Table 3 (S3): Sample concentration pre- and post-vacuum centrifugation for verification samples for the modeled linear relationship.

Pre-vacuum centrifugation (ng/µL)	Predicted concentration (ng/µL)	Post-vacuum centrifugation (ng/µL)	Time (minutes)
1.212	1.736	1.876	20
0.808	1.332	1.352	20
0.550	1.074	1.074	20
0.294	0.818	0.534	20

Supplementary Table 4 (S4): Sample volume pre- and post-vacuum centrifugation for verification samples for the modeled linear relationship.

Pre-vacuum volume (µL)	Predicted volume (µL)	Post-vacuum volume (µL)	Time (minutes)
55	33.065	32	20
55	33.065	32	20
55	33.065	32.5	20
55	33.065	32.5	20

Supplementary Table 5 (S5): Sample concentration pre- and post-vacuum centrifugation for verification samples for a linear relationship.

Sample ID	Pre-vacuum centrifugation (ng/µL)	Post-vacuum centrifugation (ng/µL)	Time (minutes)
S1.1	0.752	1.680	30
S2.1	0.728	1.300	30

S3.1	0.728	1.720	30
S1.2	0.508	1.480	35
S2.2	0.468	1.320	35
S3.2	0.450	1.240	35
S1.3	0.180	1.032	35
S2.3	0.198	1.112	35
S3.3	0.176	0.976	35
S4.1	0.534	1.890	35
S5.1	0.362	1.360	35

Supplementary Table 6 (S6): Overview of identified variants including locus, coverage, and allele frequency. Coverage is put to the upper limit of 2000; thus, coverage may be higher.

Sample	Locus	Genotype	Genes	Coverage	Allele Frequency %	concentration
S1.0	chr7:55259485	C/T	EGFR	2000	49.00	10.0
S1.0	chr12:25398284	CC/CA	KRAS	1991	38.67	10.0
S1.1	chr7:55259485	C/T	EGFR	2000	51.70	0.20
S1.1	chr12:25398284	CC/CA	KRAS	1997	40.61	0.20
S1.2	chr12:25398284	CC/CA	KRAS	1996	38.48	0.50
S1.2	chr7:55259485	C/T	EGFR	2000	48.55	0.50
S1.3	chr12:25398284	CC/CA	KRAS	1999	37.97	0.75
S1.3	chr7:55259485	C/T	EGFR	2000	51.30	0.75
S2.0						10.0
S2.1						0.20
S2.2						0.50
S2.3						0.75
S3.0	chr7:140453133	TTCA/TTCT	BRAF	1982	5.85	10.0
S3.0	chr3:178936091	G/A	PIK3CA	2000	5.00	10.0
S3.1	chr7:140453133	TTCA/TTCT	BRAF	1984	4.44	0.20
S3.1	chr3:178936091	G/A	PIK3CA	2000	5.30	0.20

S3.2	chr7:140453133	TTCA/TTCT	BRAF	1993	4.87	0.50
S3.2	chr3:178936091	G/A	PIK3CA	1999	5.10	0.50
S3.3	chr7:140453133	TTCA/TTCT	BRAF	1987	4.73	0.75
S3.3	chr3:178936091	G/A	PIK3CA	2000	5.30	0.75
S4.0	chr17:7578221	TTC/T	TP53	1976	29.5	1.4
S4.1	chr17:7578221	TTC/T	TP53	2815	25.12	0.36
S5.0	chr13:32913147	G/A	BRCA2	1997	12.42	3.96
S5.0	chr17:7577121	G/A	TP53	2000	27.15	3.96
S5.0	chr16:3832688	G/C	CREBBP	1987	16.41	3.96
S5.0	chr13:32914926	A/G	BRCA2	1995	14.19	3.96
S5.0	chr13:32913171	G/C	BRCA2	1993	14.6	3.96
S5.0	chr9:139438473	C/G	NOTCH1	1972	51.52	3.96
S5.0	chr9:98238313	C/T	PTCH1	1032	34.3	3.96
S5.0	chr2:212566773	G/T	ERBB4	1997	12.17	3.96
S5.0	chr2:16082387	C/A	MYCN	810	13.7	3.96
S5.0	chr2:16082383	G/C	MYCN	812	13.67	3.96
S5.1	chr2:212566773	G/T	ERBB4	2000	5.2	0.53
S5.1	chr9:98238313	C/T	PTCH1	469	18.76	0.53
S5.1	chr9:139438473	C/G	NOTCH1	935	34.12	0.53
S5.1	chr13:32913147	G/A	BRCA2	2000	3.9	0.53
S5.1	chr13:32913171	G/C	BRCA2	2000	4.06	0.53
S5.1	chr13:32914926	A/G	BRCA2	2000	3.72	0.53
S5.1	chr16:3832688	G/C	CREBBP	2000	7.28	0.53
S5.1	chr17:7577121	G/A	TP53	2000	11.55	0.53
S5.1	chr2:16082383	G/C	MYCN	329	6.38	0.53
S5.1	chr2:16082387	C/A	MYCN	329	6.38	0.53
